# Parental Shading Regulates Subsequent Seed Germination

**DOI:** 10.3389/fpls.2021.748760

**Published:** 2021-11-08

**Authors:** Lei Wang, Umashankar Chandrasekaran, Xiaofeng Luo, Shaowei Wei, Kai Shu

**Affiliations:** ^1^School of Ecology and Environment, Northwestern Polytechnical University, Xi'an, China; ^2^Research & Development Institute of Northwestern Polytechnical University in Shenzhen, Shenzhen, China

**Keywords:** gibberellic acid, abscisic acid, seed germination, phytohormone, parental shading

## Introduction

Seed germination is essential for subsequent young seedling establishment. Numerous elegant studies have documented the regulatory mechanisms underlying seed germination, especially phytohormones and environmental cues-mediated cascades (Shu et al., 2016; Tognacca and Botto, 2021). The promotion effect of phytohormone abscisic acid (ABA) and the repression effect of gibberellin (GA) on seed germination are extensively detected and well-documented (Shu et al., 2016, 2018). Furthermore, diverse environmental factors are also involved in seed germination control, including temperature, light, salinity, and drought. It is noted that most research about seed germination is mainly focused on the roles of these endogenous and/or environmental cues specifically during the seed imbibition stage (Shu et al., 2013, 2016; Luo et al., 2021). Whereas few studies into the effects of exposure of the parental plants to some environmental cues on subsequent seed germination processes have been published.

Among the diverse environmental factors influencing seed germination, light is attractive, which not only acts as an energy resource, but also the molecular signal for initiating seed germination (Wang and Lin, 2020). It is known that close planting leads plants to perceive the shade signal, characterized with the decrease of blue light intensity and the red (R): far-red (FR) ratio, caused by neighboring plants (Keuskamp et al., 2011, 2012; de Wit et al., 2012; Jiang et al., 2019; Zhang et al., 2019; Yang et al., 2020). In close planting conditions, competition for light triggers the plant shade response, including the change of flowering time, promotion of stem and petiole elongation, regulation of seed maturation, variation in photosynthetic response, and decrease of crop productivity (Kurepin et al., 2006; Jha et al., 2010; Elwell et al., 2011; Baker et al., 2018; Chai et al., 2018; Pantazopoulou et al., 2021). Thus, plant shade response plays a key role during the plant life cycle, and is especially important for modern agricultural production. In this research field, the effect of shade signaling on seed development and dormancy/germination are interesting and worthwhile projects, and recently some progress has been achieved. For instance, our previous study showed a higher germination rate in seeds developed under shade conditions compared to the control group, by mediating the biosynthesis of pro-anthocyanidins, fatty acids, and phytohormones ABA and GA (Chen et al., 2020). Indeed, except for the shading signal (Contreras et al., 2008), other maternal environmental cues, such as temperature, are also involved in subsequent seed dormancy and germination regulation (Kvaalen and Johnsen, 2008; Postma and Agren, 2015). Here, we concisely summarized the current understanding of parental shade-meditated seed biology, focusing on the regulatory roles of parental shading in subsequent seed dormancy and germination.

## Parental Shading Modulates Phytohormone Balance in Developing Seeds

There is a large amount of evidence reporting that diversity in phytohormones is involved in plant shade response (Sellaro et al., 2012; Sessa et al., 2018). *Arabidopsis* seeds under shade conditions show a reduction in germination with a significant increase in ABA and 12-oxo-phytodienoic acid (OPDA) content (Barros-Galvão et al., 2019). Further, an earlier study showed that seeds matured under FR light have an increased thermo-inhibition and photo-sensing capacity as well as ABA level, which in turn affect subsequent seed germination (Contreras et al., 2009). Blue light receptor CRYTOCHROME1 (CRY1) enhances seed dormancy by increasing the accumulation of ABA in *Arabidopsis* seeds under blue light-rich conditions (Barrero et al., 2014). These studies highlighted the important effect of parental shading on subsequent seed dormancy and germination control.

**Figure 1:**
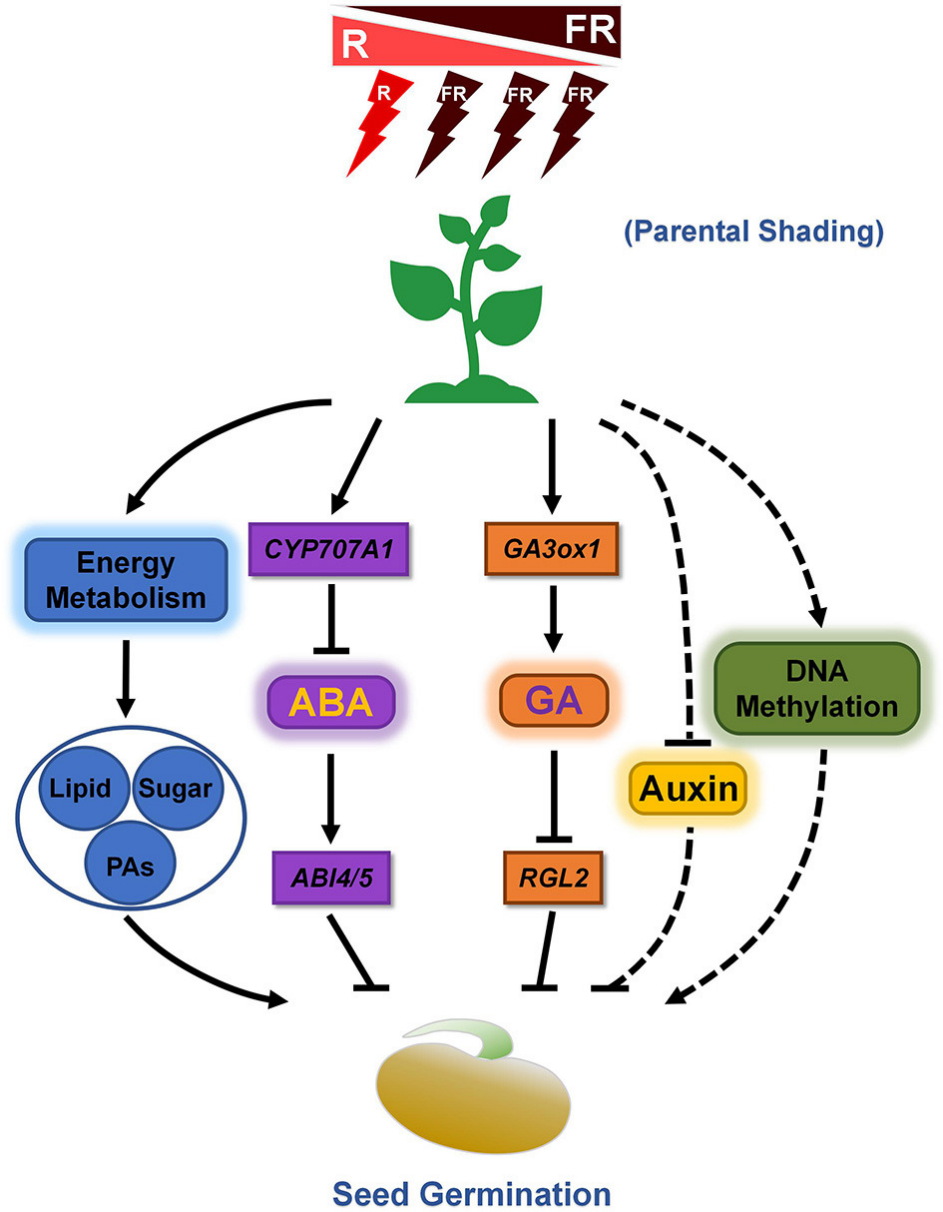
Proposed effects of shading of the mother plant on subsequent seed germination. Parental shading environment regulates seed germination by mediating the biosynthesis and signaling cascade of key phytohormones (ABA and GA), TAG content, and concentration of several types of fatty acid, sugars, and pro-anthocyanidins (PAs) in soybean (Chen et al., 2020). Furthermore, parental shading may also influence the DNA methylation level of some key genes which are involved in seed germination control in *Polygonum persicaria* (Baker et al., 2018), while the detailed mechanisms need to be further explored. In addition, given the promotion roles of auxin on seed dormancy (Liu et al., 2013), it is strongly suggested that auxin biosynthesis and signaling play some uncovered role in parental shading-mediated subsequent seed germination.

R light enhances seed germination through inhibition of the expression of ABA biosynthesis genes, while FR light delays seed germination by promoting the transcription of ABA biosynthesis genes (Barrero et al., 2014). Our previous study demonstrated that soybean seeds matured under parental shading show an increased germination rate, supported by an increase in endogenous GA content and a decrease in ABA levels, and consistently the expression level of genes involved in ABA biosynthesis are downregulated in shade condition grown seeds, while the transcription levels of the genes related to GA biosynthesis are upregulated (Chen et al., 2020). Thus, the balance between ABA and GA in regulating seed germination after shading treatment is significant (Figure 1). However, a detailed molecular analysis of the balance between ABA and GA in the seeds matured under shade conditions is currently unknown. For instance, how does shade signal regulate the corresponding genes expression? What are the key transcription factors involved in this cascade? More importantly, how the ABA-dependent primary seed dormancy is released in seeds subjected to shade is a worthwhile project to be addressed.

It is interesting that the distinct effects of parental shading on subsequent seed germination in different species are documented. For instance, in lettuce, parental shading has a negative role in subsequent seed germination (Contreras et al., 2009), while the promotion effect of parental shading on seed germination was detected in soybean (Chen et al., 2020). The difference of seed storage proteins, seed size, or even evolutionary history between lettuce and soybean may cause the distinct responsiveness for parental shading signal during seed germination, but the underlying molecular mechanisms need to be further explored.

In addition to the research on cultivated crops such as soybean (Chen et al., 2020), the similar effect of parental shading on seed size and yield in native wild species has also been detected. A recent study showed that, in *Primula vulgaris*, shading in the maternal environment led to increased seed size, but the effect of parental shading on subsequent seed germination is weak (Marin et al., 2019). Except for the seed germination processes, young seedling establishment and development are also regulated by the parental shade environment probably by mediating DNA methylation modification (Baker et al., 2018), indicating that DNA methylation regulates transgenerational environmental effects between parents and offspring (Figure 1). However, the detailed epigenetic regulatory mechanisms especially underlying phytohormones-relevant information need to be further dissected.

## Parental Shading Regulates Storage Energy Resources in Developing Seeds

Seed germination is driven by the energy stored in the seed itself (Eastmond, 2004), and especially in oil-containing seeds, such as oil rape seeds, soybean, and *Arabidopsis* seeds, the hydrolysis of triacylglycerol releases glycerol and fatty acids, and then the latter are converted to sugars which fuels seed germination (Eastmond, 2006; Bachleda et al., 2017; Zhou et al., 2019). Therefore, the levels of several types of energy resources in seeds, including sugars and fatty acid, are important for seed germination processes.

A previous study demonstrated that parental shading environment influenced the concentrations of several types of sugars and fatty acids during soybean seed development, and some of which are known to be associated with seed germination regulation. For instance, the concentrations of oleic and linolenic acid decreased in shaded-development seeds, while the concentration of linoleic acid increased, which is consistent with the faster germination phenotype of shaded-development seeds (Chen et al., 2020). Furthermore, given the repression effects of pro-anthocyanidins in seed germination (Jia et al., 2012), parental shading treatment also downregulated the levels of soluble pro-anthocyanidins in developing seeds, which further enhanced subsequent seed germination (Chen et al., 2020). Thus, the shade environment of the parent plants affects the concentration of soluble pro-anthocyanidins and several types of sugars as well as fatty acids, and finally control subsequent seed germination (Figure 1).

## Future Prospects

Perception and signaling of environmental changes are essential during the plant life cycle, including seed development and germination periods. Despite the abundance of the effect of shading on plant growth, information regarding the influence of parental shading on seed maturation and subsequent seed dormancy and germination are still elusive.

The effects of parental shading on the levels of ABA and GA in developing seeds were documented (Chen et al., 2020). It is noted that the other important phytohormone auxin plays key roles in seed dormancy and germination control (Liu et al., 2013), thus the molecular mechanisms of auxin in regulating seed germination after parental shading need to be further explored. Especially, investigation on auxin transport, signaling, and homeostasis during seed development under shading might dissect several unknown cascades beyond ABA and GA-mediated pathways (Figure 1).

Under parental shading conditions, what and how do the multiple photoreceptors (especially including phytochromes, cryptochromes, and phototropins) regulate seed development, subsequent seed dormancy release, and seed germination processes? Further, what are the relationships between the photoreceptors and phytohormones biosynthesis/signaling cascades under the parental shading environment? Indeed, these studies focusing on the parental effect on subsequent offspring growth and development provide good case studies for investigating cross-generational effects in plants induced by environmental cues. More importantly, we hope that the underlying genetic mechanisms using epigenetic approaches, including genomic DNA methylation and other molecular effects, and the precise mechanisms underlying the positive effect of parental shade signals on subsequent seed germination will be uncovered in the near future. The outcome of the proposed research ideas will provide valuable information to engineer seeds with resisting capacity under unfavorable environmental conditions without affecting the crop productivity.

## Author Contributions

LW, UC, and KS designed and jointly wrote this Opinion article. XL and SW provided inputs for the improvement of the article. All authors contributed to the article and approved the submitted version.

## Funding

Funding from the National Natural Science Foundation of China (32101670), the Science, Technology, and Innovation Commission of Shenzhen Municipality (JCYJ20190806154009040), and the Talents Team Construction Fund of Northwestern Polytechnical University (31020190QD007) supported the work in our group.

## Conflict of Interest

The authors declare that the research was conducted in the absence of any commercial or financial relationships that could be construed as a potential conflict of interest.

## Publisher's Note

All claims expressed in this article are solely those of the authors and do not necessarily represent those of their affiliated organizations, or those of the publisher, the editors and the reviewers. Any product that may be evaluated in this article, or claim that may be made by its manufacturer, is not guaranteed or endorsed by the publisher.
